# Extra Supplement—The Nursing Mirror

**Published:** 1889-04-27

**Authors:** 


All Contributions for this Supplement should be addressed "The Nursing Mirror," care of the Editor, 139-140, Salisbury Court, Fleet Street, London, E.C.

### En Passant.

READING FOR NURSES.—The following articles, which appear in the magazines of this month, should be specially interesting to nurses, though we cannot say they come exactly under the head of "nursing questions": *Murray's Magazine*, "Home Rule in the Nursery." *Cassell's*, "How to Cure Desponding," by a Family Doctor. *National*, "Brain Power of Plants," and "Women as Social Reformers." *Scribner*, "The Anatomy of the Contortionists," by T. Dwight, M.D. *The Leisure Hour*, "Philanthropic Perplexities," Harry Jones. *Fortnightly*, "Women's Suffrage Bill."

HYPNOTISM.—The medical pretensions of hypnotism have given it an importance in the eyes of the public which it did not deserve. There is really nothing so remarkable or novel in the fact that a strong mind should control a weak one, that any new proof of it should cause special excitement. Everyone knows that it is better for a patient that his doctor should inspire him with confidence, and that his nurse should be bright and cheerful; and when that is said the whole case for hypnotism is stated. Those who are well strengthen those who are sick—give them, as it were, some of their vitality. And, on the other hand, the practice of this new notion is apt to lead to mischief. The hypnotised subject is apt to become hysterical; the hypnotiser becomes demoralised by his power; and, so far as experience yet goes, the therapeutic value of the treatment is *nil*. Meanwhile, public exhibitions of hypnotism have become an unwholesome excitement, which are now forbidden in Belgium, and, it is to be hoped, will soon cease in France.

REGISTRATION OF MIDWIVES.—While Mrs. Gamp has been successfully expelled from every other part of the nursing world, she still holds her own in that department to which the good lady preferred to devote herself, with results most disastrous to mothers and children. It is calculated that among the working classes seven out of every ten children are born without the presence of a medical man. The mothers are attended by midwives who may be attentive and skilful—and we would not deny that skill comes by practice even without scientific training—but who may, on the other hand, be incompetent and cruel. As things are at present any woman that chooses may call herself a midwife; there is no proof demanded of her that she is fit for the duties she undertakes. Midwifery is a branch of medical practice that is in a special degree woman's province; and an increase in the number of trained and skilful midwives would be a national blessing. But it is imperatively necessary that no woman should be allowed to call herself a midwife, and have the lives of mothers and children entrusted to her care, who is not fit for the work she attempts. In no other country of Europe is it permitted, and it should not be permitted here. In 1878 Parliament enacted that no one should practise as a dentist who did not hold a diploma, granted after examination of his efficiency. In 1883 a similar enactment was passed with regard to veterinary surgeons. But as yet there is no register of midwives; no protection to English mothers. A meeting of ladies is shortly to be held to arouse the interest of Parliament and the public in this important matter. Miss Wilson, 45, Colville-gardens, Bayswater, will be glad to hear from ladies who desire to be present, or to learn particulars of this serious question.

THE CLOTHING OF NEW-BORN CHILDREN.—Mothers and nurses have generally had pretty much their own way in the matter of dressing babies, but the doctors are beginning to venture an opinion and a protest. From the Far West comes the condemnation of the "binder." Dr. Ady, in the *Pacific Medical and Surgical Journal*, gives it as his opinion that the binder is "a relic of barbarism, uncomfortable and mischievous, often causing, and never preventing, hernia." He advises that all "bands, skirts, etc., that punish the baby be left off." This, especially the grandly vague "etc.," is what Dr. Ady's compatriots would call "a large order," and though it is something to know what we ought to leave off in the clothing of babies, it would be much more useful to know what, in Dr. Ady's opinion, we ought to put on.

A CORRESPONDENT sends us the following hymn, written by the late Canon Kingsley, for the opening of a new wing of the Children's Hospital at Birmingham in 1870, when it was sung by 1,000 school children. It certainly ought to be incorporated among hospital literature, as being eminently suitable for any such occasion.

Accept this building, gracious Lord,
  No temple though it be;
We raised it for our suffering kin,
  And so, good Lord, for Thee.

Accept our little gift, and give
  To all who here may dwell
The will and power to do their work,
  Or bear their sorrows well.

From Thee all skill and science flow,
  All pity, care, and love,
All calm and courage, faith and hope.
  Oh, pour them from above.

And part them, Lord, to each and all,
  As each and all shall need,
To rise, like incense, each to Thee,
  In noble thought and deed.

And hasten, Lord, the perfect day,
  When pain and death shall cease;
And Thy just rule shall fill the earth
  With health, and light, and peace.

When ever blue the sky shall gleam,
  And ever green the sod;
And man's rude work deface no more
  The Paradise of God.

A DEFENCE OF ASYLUM NURSES.—"Hope" writes: "In the *Hospital* for March 30 'T. A.' asks if it would be easy to find women of refinement willing to undertake asylum work?" Yes; and in one of the largest asylums in a Midland county the majority of the nurses are refined and educated girls, who entered upon the work straight from their homes or schools. Of course, there are some very disagreeable duties to do in an asylum for the insane, and those employed hear and see very objectionable things. Still, if they only consider that the insane are rarely answerable for their actions, it is not ha f so painful as what many poor governesses have to put up with from their employers on the one hand, and the servants on the other. A great deal has been written about the heroism, etc., of a hospital nurse, and little of that of the asylum nurse. Indeed, *The Hospital* is the only paper in which I have seen a kind or favourable word for the latter. If the truth was known, it would be found there is a great deal more heroism in asylums than in hospitals. It is in very few cases that a hospital nurse's life is in danger, while that of the asylum nurse is not safe during the whole time she is on duty. Perhaps, if the latter aired her grievances in the newspapers she would meet with more sympathy and less obloquy.

### Sick Children in their Homes.

INVALID CHILDREN'S AID ASSOCIATION, 18, BUCKINGHAM-STREET, STRAND, LONDON, W.C.

We put the name and address of the society at the head of our remarks because in this instance all depends on the address being remembered. The root-idea of this association is that it must be useful for all who have to do with the suffering poor, especially for medical men, matrons of hospitals, and nurses, to know of one central spot to which they can send particulars of any invalid or crippled child that needs befriending. It matters not what the child requires—it may be befriending simply, that is to say, the constant visits of a kindly and intelligent lady, who is expected to devote herself a great deal to her child, and to make its life very different from what it was before. Or a folding spinal carriage may be needed, so that the patient may get what is otherwise unattainable—a little fresh air. Or a trained nurse may be required to pay daily or occasional visits to dress wounds, adjust appliances, etc. Or the child should be sent away from its own home, too often wretched and poverty-stricken, to a convalescent or incurable institution. It is the aim of the association to supply whatever the sufferer needs, so far as it is possible and reasonable to provide it.

The chief feature of the work is the Visitor. The first step taken at the central office usually is to select one of the ladies on the list, and ask her to become the *friend* of the child in question. From that moment she becomes, as regards that particular case, the *aide-de-camp* of the committee. She informs herself of the child's circumstances and needs, and reports on them to the office. If occasion requires she accompanies the child to the hospital, so that the doctor's instructions may be given to one competent to understand them, which the mothers too often are not. If special diet is wanted, she often makes it her business to provide it. And often, when the benefit needed involves much expense, she helps the committee by mentioning the matter amongst her own friends, and thus raising a part of the sum required. It is specially wished that ladies willing to share in this work should send their names to the Hon. Secretary of the association. It need not involve the befriending of more than one afflicted child, and is thus open to many who cannot undertake regular district visiting. The office expenses are kept as small as possible, but the expenses incurred for the children are sometimes considerable, and subscriptions will be thankfully acknowledged by the hon. treasurer, Mrs. Warrington Haward, 16, Savile-row, W.

### Patients, not Prisoners.

The *Nineteenth Century* for this month contains an article by Dr. Batty Tuke entitled "Lunatics as Patients, not Prisoners." Dr. Tuke pleads for the establishment of lunatic hospitals rather than asylums, and the use of all possible means to cure insanity by the physician in charge. It seems almost unnecessary to make such a plea, and yet it is most needful. To have been in an asylum and to have been in prison are alike things spoken of with bated breath, and many a good and clever man whose nervous system—as might another's muscular system—has been exhausted by overstrain has his whole future life and work darkened and disparaged by the phrase, "He was once in an asylum." Moreover, as things are at present, recovery in an asylum is very much a matter of chance. The absence of outside worries may bring it about, but scientific and consciously intended treatment is not encouraged. If the asylum were kept as a place of abode for lunatics with whom the hospital treatment had failed, it would fulfil its mission better, and there would be less demand for its accommodation. This is a question on which Dr. Tuke has a right to speak. The asylum of which he is the head has shown most encouraging results, due in great part to the use of the "open-door" system, which wakes the dormant moral sense of the patients, and to the employment of educated and refined ladies as attendants, or rather companions, giving the patients the intellectual companionship they need.

### Everybody's Opinion.

[*Correspondence on all subjects is invited. No communications can be entertained if the name and address of the correspondent is not given, or unless one side of the paper only be written on.*]

### WHITE SLAVES!

Veritas writes: Reading the article in the *Pall Mall Gazette* on "White Slavery in Hospitals," I awoke to the fact of how little some people know about nurses, and how few "outside people" understand them. There has been a good deal of discussion about nurses, but it is carried on by people who do not know their ways. The nurses have remained silent, save where here and there one of the discontented ones has given a great deal of abuse to the particular hospital where she was trained, or—more likely—dismissed at the end of her first month. Perhaps, through "tact," she may have put in her first year before it was discovered that she never would make a nurse.

The majority of nurses dislike to be brought before the notice of the public. They choose their profession from personal reasons. There are others open to them certainly; but if they choose to be nurses, and not governesses, clerks, and domestics, who has the right to interfere if they do not grumble? I emphatically deny that nurses are foolish. Brave, noble, and self-sacrificing they may be—often they are; but they are strong-minded and would speak out at once if they felt themselves aggrieved, and would not "suffer in silence" any indignity. I look upon the years I spent in hospital as the happiest, because the most useful, of my life. There I met people, even amongst the patients, the like of whom I have never met outside. I have known nurses, who have had a private income, who have worked for remuneration on an equal footing with a nurse who had lived in domestic service, sisters in one profession; and never by word or deed was any difference shown in training of either, though the one was of a noble, truly noble, family, and the other was a woman earning a livelihood, and doing what good lay in her power at the same time. Many of the nurses do not labour of necessity. They are of good family, and have money which they spend liberally, but you might live amongst them for years and not know it.

From the patients, the nurses I know and myself always got civility, and a sort of nurse-worship which has a tendency to make us conceited, from the doctor's kindness and courtesy. The only grievance we have to complain of is the society of the town. It would teach a lesson to those ladies who visited the hospital and speak patronisingly to the nurses to hear a nurse's opinion of them. They are the people who are insolent to the nurses, and whom the nurses heartily despise. They will even advise a nurse as to the treatment of her patients.

A nurse will forgive her patients not treating her as a lady, but they never forgive people who, not being so well bred as themselves, dare to speak scandal of them. These are the people who, over their afternoon tea, speak of nurses flirting with doctors and patients, and are disgusted when a doctor marries a nurse, even if the nurse's position was superior to the doctor's. These are the people who continually remind you they are subscribers. Naturally, we do not like them. We nurses do not want pity, but we do claim justice and esteem.

### Appointments.

Mrs. Alice Parry, late sister at the Royal Hospital, Waterloo-bridge-road, has been appointed matron of the Royal Hospital, Weymouth.

### Vacancies.

The following vacancies are announced:

**Nurses.**

Institution of Trained Nurses, Leicester (four).

Birmingham Infirmary, Dudley-road (several), £25.

City Scarlet Fever Hospital, Birmingham (Two).

Carmarthenshire Infirmary, £18.

Eartbourne Nursing Inst. (several).

Kendal Hospital, £28.

Princess Alice Hospital, Eastbourne £28.

Royal Hospital for Children and Women, S.E.

Newport and County Infirmary.

St. Cuthbert's Poor House, Edinburgh, £25.

Kingston Nurses' Home, Hull.

Jersey Nurses' Inst. (two), £25.

Rochdale Infirmary, £22.

Kent Nursing Inst. (several).

Cottage Hospital, Elham.

Windsor Royal Infirm., £20 to £25.

